# Dr. J. B. Brinton’s Case of Puerperal Convulsions

**Published:** 1842-05-28

**Authors:** J. B. Brinton

**Affiliations:** West Chester, Pa.


### *Case of Puerperal Convulsions.*

By J. B. Brinton, M. D.

To the Editors of the Medical Examiner.

Gentlemen,—I send you a short notice of a case of "Puerperal Convulsions" which occurred in my practice. If you deem it of sufficient interest to fill a page in your excellent journal, I place it in your hands.

On the 14th of last February, at 8½ A. M., I was called in consultation to Mrs. L——, aged 23 years, in the ninth month of her pregnancy, with her first child. The physician in attendance informed me that she had been attacked with convulsions the day before at noon; that they had continued up to the present, leaving her entirely insensible during the whole time; bled forty ounces during the early part of the attack. Upon my going to the bed side I found her of a very full and plethoric habit, entirely comatose, with an active, full, and frequent pulse, skin hot, respiration greatly impeded, loud and stertorous, considerable suffusion of the eyes and face, *pupil contracted*, face swollen and features distorted, and in a few minutes a violent convulsion ensued, which required the aid of four or five persons to render her secure. The *os tincæ* was very little dilated, rigid, tumid and unyielding. I immediately bled her fifty ounces from a large orifice, which soon rendered the pulse soft and compressible, and I had the gratification at 10½ A. M. to find the parts sufficiently dilated to admit of the application of the forceps; the situation of the head was favourable, and I effected the delivery with but little difficulty. Previous to the use of the instruments there was a suspension of the pains, and the *secale* ceased to produce its accustomed effect. She had in all eighteen paroxysms, three of which occurred after delivery. As they continued of a very threatening character, ice was applied to the head, six ounces of blood from the same by cups, blister to the nape of the neck, and stimulating enemata.

After these measures she had not a return of the convulsions, but remained altogether insensible till eleven o'clock at night, when she showed a partial return of sensibility by making some domestic inquiries. Upon my second visit, on the 15th, I found considerable aberration of mind, but symptoms favourable to recovery; her restoration to health was speedy. I would add, this is another instance of the successful treatment practised and recommended by the much lamented Dewees.

*West Chester, Pa., 20th May,* 1842.

